# Comprehensive Analysis of Correlations in the Expression of miRNA Genes and Immune Checkpoint Genes in Bladder Cancer Cells

**DOI:** 10.3390/ijms22052553

**Published:** 2021-03-04

**Authors:** Przemysław A. Stempor, Dror Avni, Raya Leibowitz, Yechezkel Sidi, Maria Stępień, Tomasz Dzieciątkowski, Paula Dobosz

**Affiliations:** 1 SmartImmune Ltd, Accelerate Cambridge, University of Cambridge Judge Business School, Cambridge CB4 1EE, UK; pstempor@gmail.com; 2 Laboratory of Molecular Cell Biology, Center for Cancer Research and Department of Medicine C, Sheba Medical Center, Tel Hashome 52621, Israel; droravni@msn.com; 3 Oncology Institute, Shamir Medical Center, Be’er Yaakov, Tel Hashome 52621, Israel; rayal@shamir.gov.il; 4 Faculty of Medicine, Sackler School of Medicine, Tel Aviv University, Tel Aviv-Yafo 6997801, Israel; yehezkel.sidi@sheba.health.gov.il; 5 Faculty of Medicine, Medical University of Lublin, 20-059 Lublin, Poland; mmaria.stepien@gmail.com; 6 Department of Microbiology, Medical University of Warsaw, 02-005 Warsaw, Poland; dzieciatkowski@wp.pl; 7 Department of Hematology, Transplantationand Internal Medicine, Medical University of Warsaw, 02-097 Warsaw, Poland

**Keywords:** miRNA, microRNA, bladder cancer, noncoding RNAs, immune checkpoints, immunological synapse

## Abstract

Personalised medicine is the future and hope for many patients, including those with cancers. Early detection, as well as rapid, well-selected treatment, are key factors leading to a good prognosis. MicroRNA mediated gene regulation is a promising area of development for new diagnostic and therapeutic methods, crucial for better prospects for patients. Bladder cancer is a frequent neoplasm, with high lethality and lacking modern, advanced therapeutic modalities, such as immunotherapy. MicroRNAs are involved in bladder cancer pathogenesis, proliferation, control and response to treatment, which we summarise in this perspective in response to lack of recent review publications in this field. We further performed a correlation-based analysis of microRNA and gene expression data in bladder cancer (BLCA) TCGA dataset. We identified 27 microRNAs hits with opposite expression profiles to genes involved in immune response in bladder cancer, and 24 microRNAs hits with similar expression profiles. We discuss previous studies linking the functions of these microRNAs to bladder cancer and assess if they are good candidates for personalised medicine therapeutics and diagnostics. The discussed functions include regulation of gene expression, interplay with transcription factors, response to treatment, apoptosis, cell proliferation and angiogenesis, initiation and development of cancer, genome instability and tumour-associated inflammatory reaction.

## 1. Introduction

Traditionally, cancer patients have been prescribed their medications based on the tissue of origin, but also symptoms and signs of their disease, according to the rule “one size fits all” [1]. Personalised medicine is a leading medical model proposing customisation of healthcare [1,2]. According to this strategy, treatment and all medical procedures should be tailored to the particular patient, especially to their genetic makeup, not only the disease. Thus, stratified medicine is a concept based on identifying subgroups of patients having distinct characteristics, mechanisms of disease or responses to treatment [3].

Not only the treatment is tailored in this concept, it also requires very precise diagnostic tests, able to identify a subgroup of patients which will benefit from the treatment or those patients whose health might be at risk when given certain drugs. Particularly prominent diagnostic procedures include genomics-based molecular methods, with the aid of imaging and analytical/bioinformatical tools. Development of molecular personalised medicine requires interdisciplinary teams composed of medical and biological specialities. It requires a good understanding of human organism complexity, environmental exposures, genomic interactions and the disease process itself [1].

Unfortunately, precision medicine is being implemented very slowly in cancer management. The need for good biomarkers is more urgent than ever. For example, platinum-based chemotherapy and/or cystectomy remain the major clinical interventions undertaken for bladder cancer patients. However, the only factors considered for the selection of therapy are the disease progression and responsiveness to chemotherapy [4,5]. Given the incredible progress in immunotherapy, the need for clinically useful biomarkers and molecular methods for patient stratification is obvious, as only approximately 50% of the patients respond to chemotherapy. Another group responds well to checkpoint inhibitors (in most cases 15–30%; depending on the drug and trial, it can be up to 46%) to give some examples [4].

MicroRNAs (miRNAs, miRs) are abundant small non-coding RNAs composed of 22–24 nucleotides [6,7]. They play an important role in post-transcriptional gene suppression [8] and have been reported to be involved in cellular processes, such as differentiation, morphogenesis and tumorigenesis [8,9]. miRNAs usually target the 3’unranslated region (UTR) of the mRNA and less frequently the 5’UTR or the coding sequence of their target mRNA [9]. Thus, miRNAs regulate gene expression, and each miRNA molecule targets tens to hundreds of mRNAs [6]. Furthermore, some mRNA targets might be combinatorically affected by several different miRNA molecules, increasing the complexity and precision of post-transcriptional regulation, and fine-tuning the expression level of genes [6]. Aberrant miRNA expression is found in a variety of cancers, including bladder cancer, suggesting that they may have roles as oncogenes or tumour-suppressor genes [8]. Half of all the miRNA genes are located in genomic regions known to be associated with cancer or in fragile sites often altered in human cancers [9]. These observations clearly show the importance of miRNAs function in cancer.

Selected groups of specific miRNAs are commonly altered in particular cancers, and recent data have shown that miRNA expression profiles of tumours are able to discriminate between different types of cancer [10]. Thus, miRNA profiles might be more useful for cancer diagnosis and prognosis [11], whereas profiles based only on mRNA have been proven to be generally unreliable, to date [10]. Several miRNA:mRNA interactions have been proven to be important for cancer pathogenesis so far, with let-7 miRNA family being the most well-studied [12]. The family of miR let-7 regulates RAS oncogenes, among others, and the expression of let-7 is reduced in several tumours, including lung cancer [12].

At the moment, there are no reliable molecular biomarkers for bladder cancer prognosis, treatment, response or progression, despite advances achieved through novel checkpoint therapies [13,14]. Significant effort has been invested in unravelling predictive biomarkers for the response to immune checkpoint inhibition in many cancers, including bladder cancer, melanoma or lung cancer [13,15]. PD-1/PD-L1 expression in tumour cells and in the tumour microenvironment, genetic alterations, mutational load in tumour cells, epigenetic changes, miRNAs expression, pre-existing immunity and its enhancement during treatment by the tumour-infiltrating immune cells were associated with better outcomes and were shown to be predictors for immune checkpoint inhibition. However, further studies are required to assess their predictive power and facilitate the implementation into the clinic [13,15]. In this paper, we present results from a comprehensive bioinformatical analysis of the correlation between the expression of 21 genes known to be involved in the immunological synapse and miRNAs in bladder cancer. We also added three transcription factors discovered in our previous work [16] to be positively correlated with the expression of selected checkpoint mRNAs from the network we previously reported.

## 2. Results

### 2.1. Correlations Analyses in Bladder Cancer Samples—Genes mRNA to miRNA

In this study, we investigated co-expression profiles of miRNAs and co-inhibitory and co-stimulatory checkpoint genes potentially involved in the immunological synapse. To facilitate this task, we visualised the correlations as both static and interactive heatmaps for the bladder cancer dataset available in The Cancer Genome Atlas (TCGA). This resource is now available publicly as an interactive website (see Supplementary Materials Section, Figures S1 and S2).

In order to create a good starting point for further investigations aiming to assess regulatory dependencies and genetic interactions between genes and microRNAs, we calculated correlations between all miRNAs and a selected set of 21 genes, known from the literature to be potentially expressed on the cancer side of the immunological synapse: CD112, CD137L, CD200, CD276, CD277, CD40, CD48, CD70, CD80, CD86, GITRL, HHLA2, HVEM, ICOSLG, LGALS9, OX40L, PD−L1, PD−L2, PVR, VISTA and VTCN1. To present a more complete picture of regulation, we also included three transcription factors (TFs), which we identified in our previous research to be correlated with the co-expressed checkpoint genes in this set: BACH2, MAFK and NFE2L2 [16].

**Figure 1:**
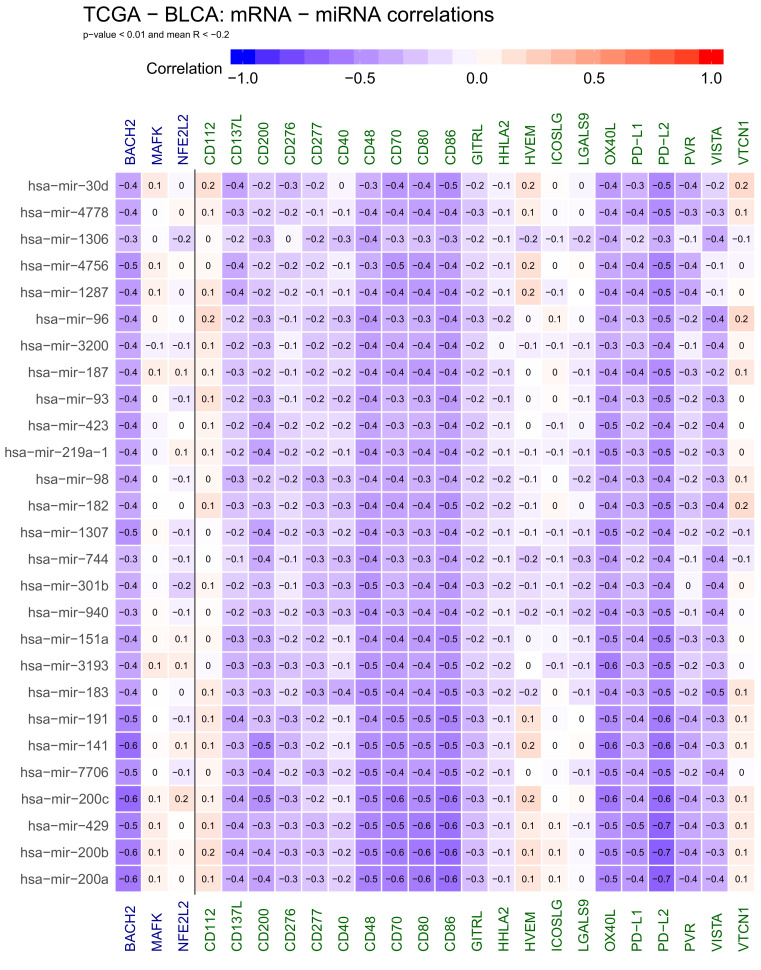
Heatmap showing negative correlations between selected genes active at cancer side of the immunological synapse: CD112, CD137L, CD200, CD276, CD277, CD40, CD48, CD70, CD80, CD86, GITRL, HHLA2, HVEM, ICOSLG, LGALS9, OX40L, PD−L1, PD−L2, PVR, VISTA and VTCN1, transcription factors: BACH2, MAFK and NFE2L2 and miRNA. Negative correlations are shown in blue. The numbers in heatmap tiles represent the correlation coefficients. The following statistical criteria were used to filter miRNA: correlation coefficient <= −0.2 for anticorrelated microRNAs and statistical significance of correlation (*p*-value from cor.test function in R) < 0.01.

We chose to further study miRNAs that are negatively correlated with gene expression under our assumptions that these may directly negatively regulate one of the genes of interest. Conversely, positive correlation might indicate indirect regulation; for example, miRNAs that negatively regulate suppressors of the genes of interest, but may also indicate more complex crosstalk. Using these criteria, we obtained 27 miRNAs generally anti-correlated with gene expression: hsa−mir−30d, mir−4778, mir−1306, mir−4756, mir−1287, mir−96, mir−3200, mir−187, mir−93, mir−423, mir−219a−1, mir−98, mir−182, mir−1307, mir−744, mir−301b, mir−940, mir−151a, mir−3193, mir−183, mir−191, mir−141, mir−7706, mir−200c, mir−429, mir−200b and mir−200a (Figure 1).

The correlation profiles of all 27 miRNAs are very similar, suggesting that they all contribute to the co-suppression of immune synapse genes. However, their correlation with genes is not uniform: CD48 CD70, CD80, CD86, OX40L and PD−L2 are most strongly anticorrelated to miRNA expression, indicating that this cluster of genes is under the strongest control. In the previous report [16], we found most of these genes to be strongly correlated with each other and possibly working together in synergy. At the same time, the results suggest the existence of a second set of genes that are not anticorrelated or weakly correlated with the set of putative downregulating miRNAs: CD112, VEM, ICOSLG, LGALS9 and VTCN1. Interestingly, we found these genes correlated with other genes in the immune response set—this intuitively suggests that not all, but only a specific subset of immune response genes is under specific miRNA control.

The TCGA database contains the expression levels of pre-miRNAs and not of the mature miRNAs. Some of the top correlated and anticorrelated miRNAs, for example, mir-199a-1 and mir-199a-2, have different pre-miRNA sequence, but produce identical mature miRNA, and are extremely likely to target the same genes. Furthermore, among our top hits, there are miRNAs, which have a different mature sequence, but share the same seed region such as mir-146a and mir-146b, and are also likely to target the same genes. However, their transcriptional and post-transcriptional regulation might be different, and they can potentially perform different functions depending on transcription of other miRNAs and coding genes. We observed identical correlation profiles for some miRNAs sharing the mature sequence (mir-125b-1 and mir-125b-2). However, the expression profiles were similar, but not identical for miRNAs, sharing only the seed region: mir-146a and mir-146b; mir-199a and mir-199b. Additionally, mir-199a−1 and mir-199a−2, despite sharing an identical sequence, had similar, but not identical correlation profiles.

**Figure 2:**
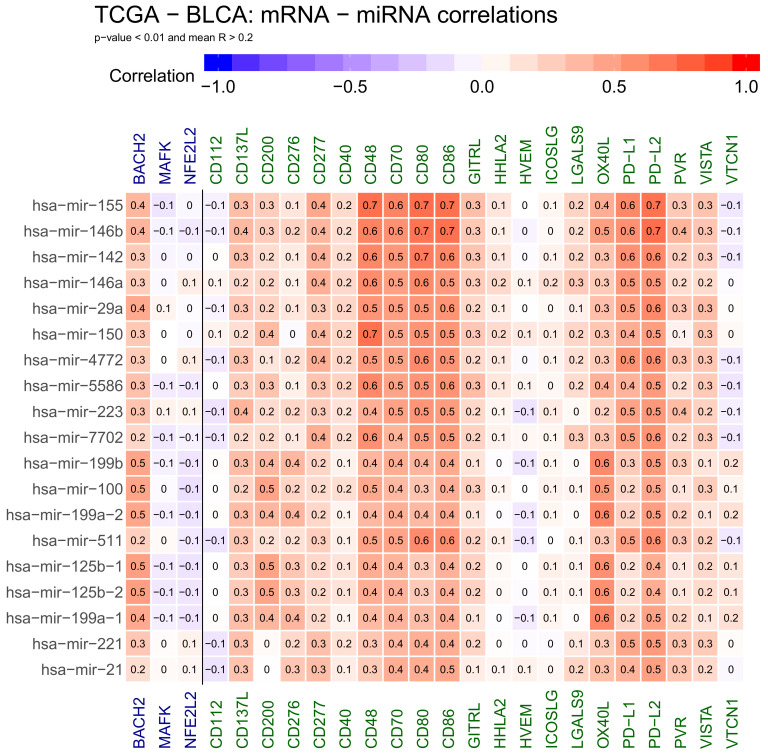
Heatmap showing positive correlations between selected genes active at cancer side of the immunological synapse: CD112, CD137L, CD200, CD276, CD277, CD40, CD48, CD70, CD80, CD86, GITRL, HHLA2, HVEM, ICOSLG, LGALS9, OX40L, PD−L1, PD−L2, PVR, VISTA and VTCN1, transcription factors: BACH2, MAFK and NFE2L2 and miRNA. Positive correlations are shown in red. The numbers in heatmap tiles represent the correlation coefficient. The following statistical criteria were used to filter miRNA: correlation coefficient >= 0.2 for correlated microRNAs and statistical significance of correlation (*p*-value from cor.test function in R) < 0.01.

We also obtained 19 miRNAs generally correlated with gene expression: mir−155, mir−146b, mir−142, mir−146a, mir−29a, mir−150, mir−4772, mir−5586, mir−223, mir−7702, mir−199b, mir−100, mir−199a−2, mir−511, mir−125b−1, mir−125b−2, mir−199a−1, mir−221 and mir−21 (Figure 2). In this case, it is more difficult to speculate if this positive correlation indicates indirect regulation or these miRNAs are generally upregulated in cancer alongside immune response genes and are responsible for different functions, for example suppressing an unrelated set of genes.

### 2.2. Correlations Analyses in Bladder Cancer Samples—Genes to Genes

**Figure 3:**
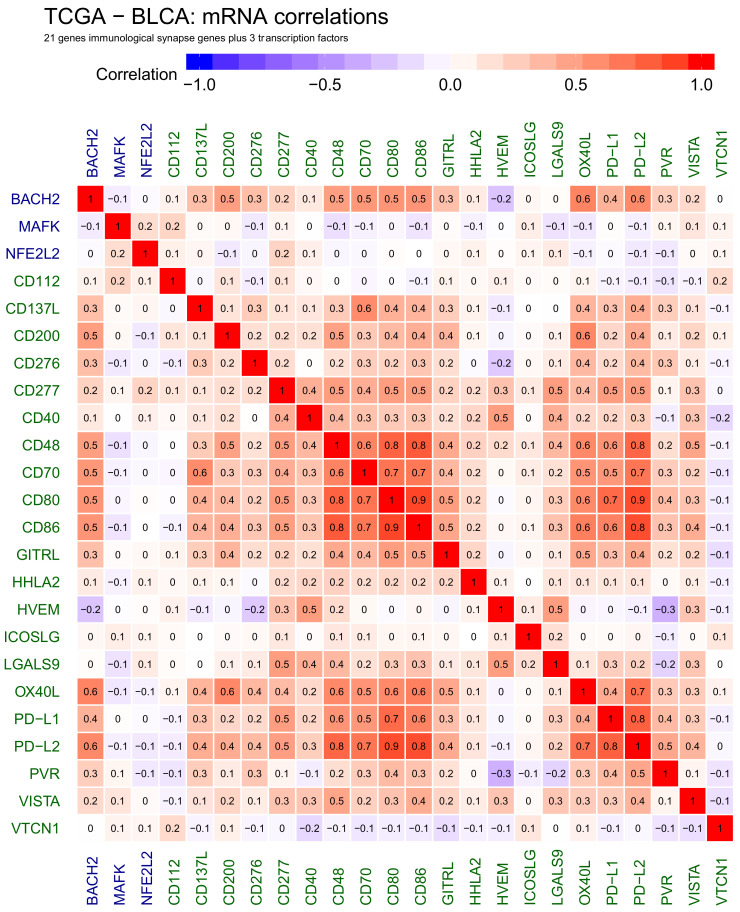
Heatmap showing correlations between selected genes active at cancer side of the immunological synapse: CD112, CD137L, CD200, CD276, CD277, CD40, CD48, CD70, CD80, CD86, GITRL, HHLA2, HVEM, ICOSLG, LGALS9, OX40L, PD−L1, PD−L2, PVR, VISTA and VTCN1, transcription factors: BACH2, MAFK and NFE2L2. Positive correlations are shown in red, negative correlations are shown in blue. The numbers in heatmap tiles represent the correlation coefficients.

**Figure 4:**
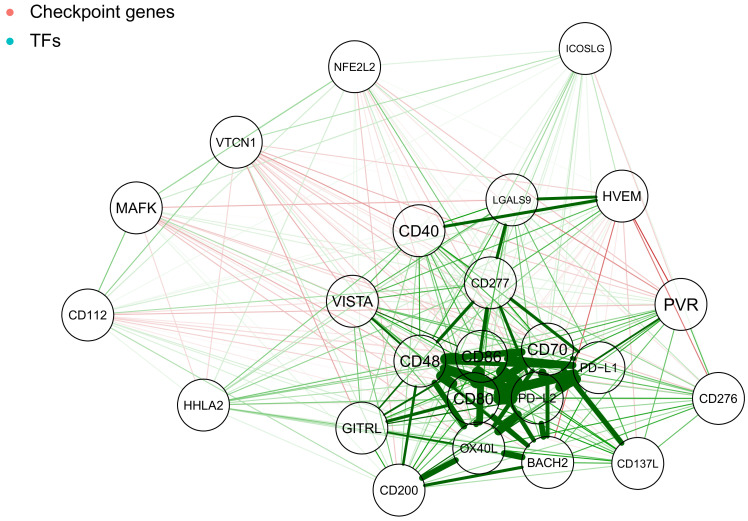
Expression correlation network showing genes active at cancer side of the immunological synapse (red nodes): CD112, CD137L, CD200, CD276, CD277, CD40, CD48, CD70, CD80, CD86, GITRL, HHLA2, HVEM, ICOSLG, LGALS9, OX40L, PD−L1, PD−L2, PVR, VISTA and VTCN1 and transcription factors (blue nodes): BACH2, MAFK and NFE2L2. Green lines represent positive correlations, and red lines represent negative ones. Line thickness represents the strength of the correlation.

To better understand the impact of underlying gene regulatory network on genes-miRNA interactions, we analysed the correlations between the genes and transcription factors only. These created a symmetric correlation heatmap (Figure 3), which we further projected as a correlation-driven network using a force-directed layout (Figure 4).

The genes that we previously observed to be well-correlated and anticorrelated to miRNA hits, CD48 CD70 CD80 CD86 OX40L PD−L1 PD−L2, show a good correlation with each other resulting in a distinctive cluster (Figure 4). Additionally, the BACH2 transcription factor is well-corelated to genes within this group. Other genes are either not that strongly correlated, or strongly correlated only to a single gene within the cluster, rather than forming further cliques.

### 2.3. Correlations Analyses in Bladder Cancer Samples—miRNA to miRNA

**Figure 5:**
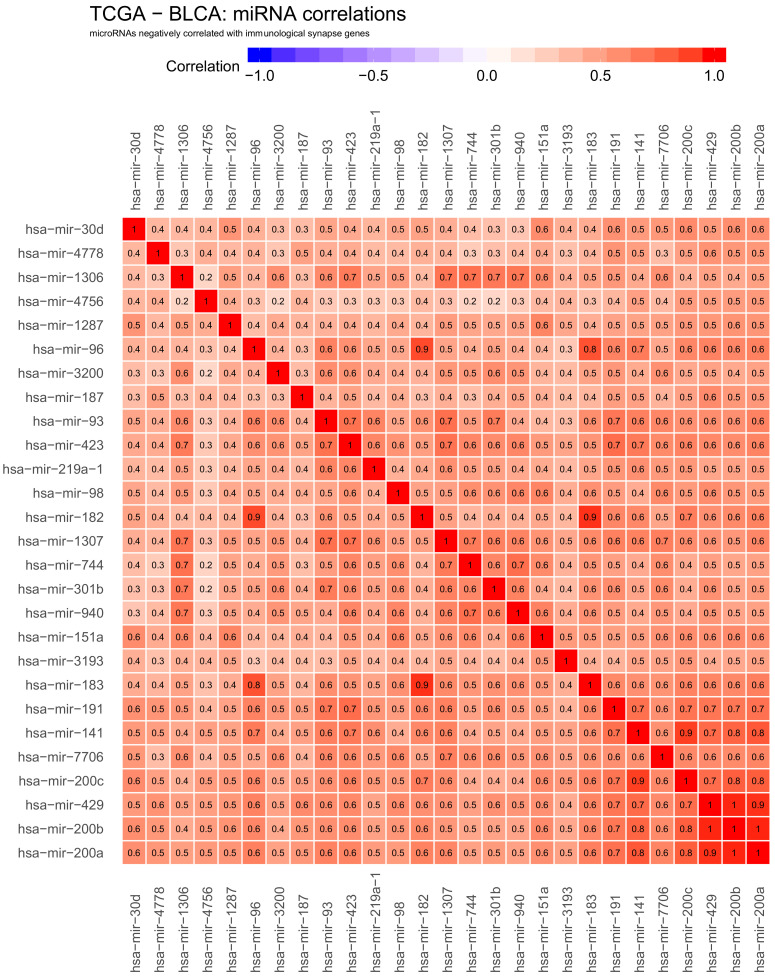
Heatmap showing correlations within miRNA hits negatively correlated to checkpoint genes. Positive correlations are shown in red, negative correlations are shown in blue. The numbers in heatmap tiles represent the correlation coefficients.

**Figure 6:**
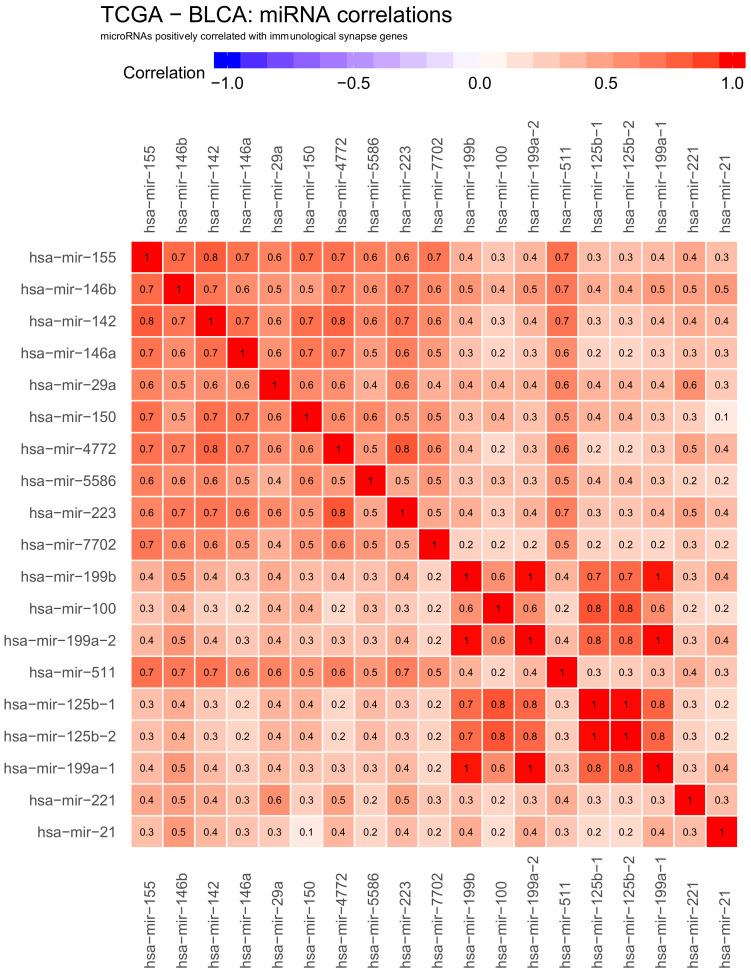
Heatmap showing correlations within miRNA hits positively correlated to checkpoint genes. Positive correlations are shown in red, negative correlations are shown in blue. The numbers in heatmap tiles represent the correlation coefficients.

**Figure 7:**
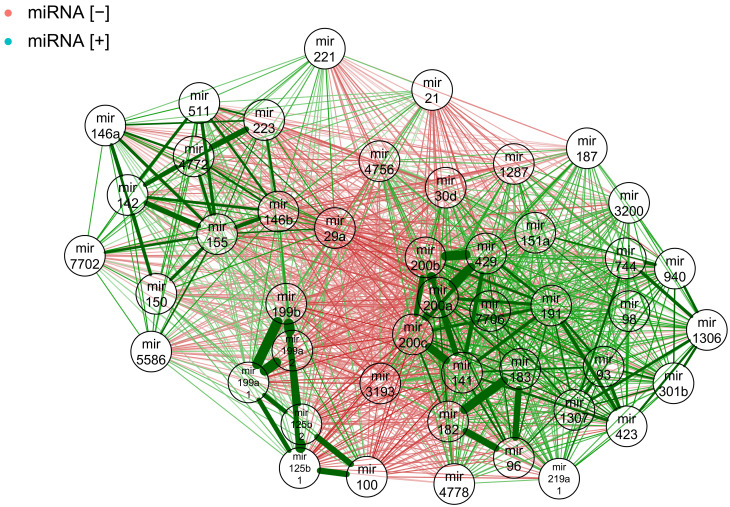
Expression correlation network showing positive (blue negative) and negative (red negative) miRNA hits. Green lines represent positive correlations and red lines represent negative ones. Line thickness represents the strength of the correlation.

To investigate if gene–miRNA interaction is influenced by a specific pattern of correlations between miRNAs themselves, we analysed the correlations within previously identified miRNA hits. Figure 5 and Figure 6 show correlations within negatively and positively correlated miRNA hits, respectively. We also projected these correlations on the common interaction network using a force-directed layout (Figure 7).

As expected, both groups of miRNAs were well-correlated within each set, with the correlation of 1 or close to one for miRNAs leading to the same mature microRNA. However, we observed some examples of nearly identical expression profiles between different miRNAs, for example, hsa−mir−429, hsa−mir−200b and hsa−mir−200a in negative hits; and hsa−mir−199b, hsa−mir−100, hsa−mir−199a−2, hsa−mir−125b−1 and hsa−mir−125b−2 hsa−mir−199a−1 in positive hits.

When visualised as a network, correlations within miRNA targets showed similar expression profiles within positive and negative hit groups. Between these two groups, correlations were negative indicating—as expected—opposite expression patterns. Interestingly, we observed a further sub-clustering within both groups. Positive hits formed two distinct clusters, while negative hits formed two smaller, tightly correlated cliques with other miRNAs more loosely correlated.

### 2.4. Network of Correlation Derived Interactions between miRNAs, Checkpoint Genes and TFs

**Figure 8:**
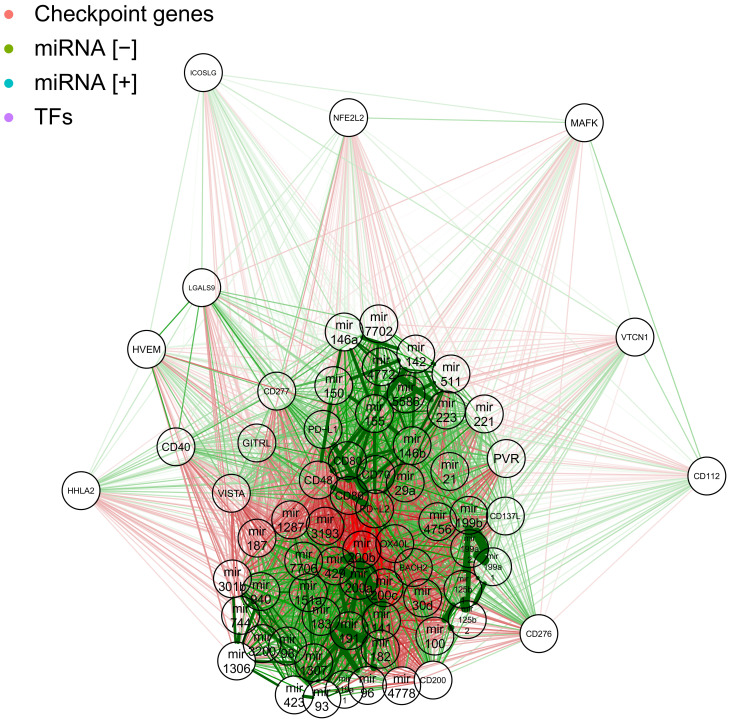
Combined expression correlation network showing negative (green nodes) and positive (blue nodes) miRNAs hits; immunological checkpoint genes (red nodes): CD112, CD137L, CD200, CD276, CD277, CD40, CD48, CD70, CD80, CD86, GITRL, HHLA2, HVEM, ICOSLG, LGALS9, OX40L, PD−L1, PD−L2, PVR, VISTA and VTCN1; and transcription factors (purple nodes): BACH2, MAFK and NFE2L2. Green lines represent positive correlations and red lines represent negative ones. Line thickness represents the strength of the correlation.

We analysed all the dependencies and possible interactions between miRNAs, checkpoint genes and TFs. We visualised them and a combined correlation network. Figure 8 shows the features we discovered in previous steps in a broader context. The gene cluster of CD48, CD70, CD80, CD86, OX40L, PD−L1, PD−L2 and BACH2 transcription factor is well-connected and visible in between positive and negative miRNA hits territories. Negative hits show well-connected cliques, that are also better anti-correlated with the cluster of checkpoint genes. Positive miRNA hits show two bigger, distinct clusters, of which one is more strongly correlated to the cluster of checkpoint genes.

### 2.5. Immune Synapse Genes Are Deregulated in Breast Cancer

**Figure 9:**
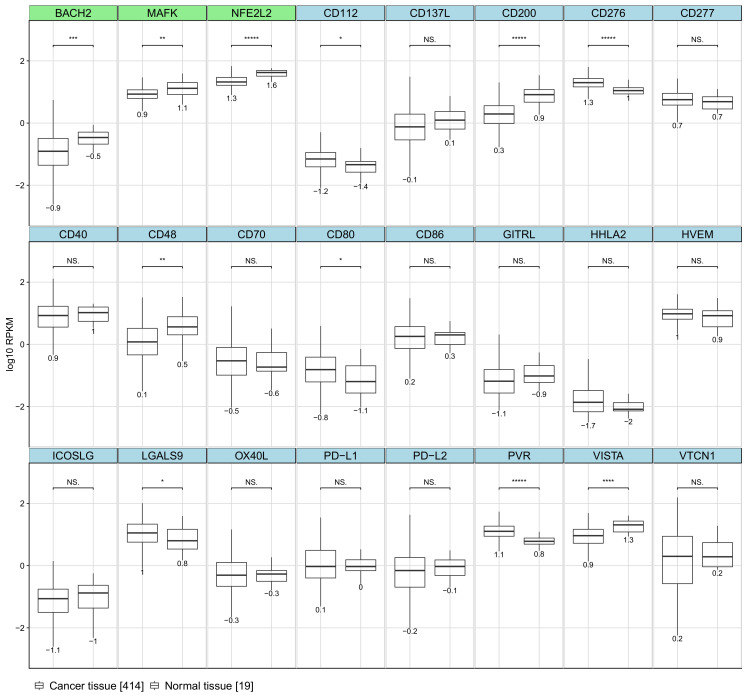
Comparison of gene expression values in cancer vs. normal tissue. Visualised values represent fragments per million reads per kilobase of transcript (FPKM) obtained from TCGA. Violin plots show the distribution of expression values, with imposed boxplots visualising box statistics. A value below the violin plot denotes the median of the sample. The stars above each group indicate the significance level (*p*-value) derived from Mann–Whitney (Wilcoxon) U test. *p*-value mapping go as follows: ***** represents a *p*-value below 0.00001, **** represents a *p*-value below 0.0001, *** represents a *p*-value below 0.001, ** represents a *p*-value below 0.01 and * represents a *p*-value below 0.1; “NS.” denotes not significant.

Finally, we analysed how the expression of the immune synapse gene set changes between cancer sample and normal tissue. It should be noted that this study is severely limited since cancer tissue data set in TCGA are much more numerous than normal tissue 414 and 19 samples, respectively. We obtained gene expression value estimates as fragments per million reads per kilobase of transcript (FPKM) and visualised their distributions with imposed box statistics in Figure 9. Direct comparison of FPKM distributions show us that all three transcription factors: BACH2, MAFK and NFE2L2 are downregulated in cancer cells. CD48, CD200 and VISTA are also downregulated in cancer samples, while CD276, LGALS9 and PVR are upregulated in cancer tissue. To understand these changes in the context of global expression, we have run differential expression analyses using the DEseq2 package. Taking into account a global dispersion and values of the expression, DEseq found MAFK, NFE2L2, CD200, CD80, HHLA2, LGALS9, PD−L1 (CD274) and PVR to be significantly upregulated, while CD112 (PVRIG), CD276, CD48 and VISTA (VSIR) are significantly downregulated. It should be noted that as with the very low expression values, such as BACH2, they are not assigned as significantly deregulated. However, analysing the distributions of the expression of BACH2 in cancer vs. normal tissue we see a small but significant difference.

## 3. Discussion

It has been suggested that bladder cancer should not be divided merely based on the characteristic of muscle invasion, thus generating two groups: muscle-invasive (MIBC, around 20% of bladder cancers at diagnosis) vs muscle-non-invasive (NMIBC, over 60% at diagnosis), based on the mutations they possess [12]. Both types have been studied extensively and it is clear that they differ genetically; for example, NMIBC has significantly fewer genomic rearrangements and mutations, whereas MIBC is characterised by frequent chromothripsis events, leading to many different aberrations [12]. Thus, currently, it is accepted that two types of bladder cancer mentioned above have several subgroups, still under investigation. It is clear if miRNAs can be used as biomarkers for these subgroups. Interestingly, most of the miRNAs analysed and described here were not previously reported to be involved in immunological synapse genes regulation. However, they were well connected with oncogenesis or cancer invasion events, as well as cancer’s response to the therapeutics.

### 3.1. The Most Positively Correlated miRNAs

The top hit in correlated miRNAs, mir−155, is a known oncogenic microRNA that targets the ELK3 transcription factor that is imperative in the response to hypoxia [17]. It was shown to be upregulated in breast cancer and linked to PARP-1 inhibitors response [18]. In bladder cancer, this miR is known to be significantly overexpressed, promoting tumour growth by repressing DMTF1, a tumour-supressing gene [19].

The second-best hit, miR-146b, is a known tumour-suppressor targeting TRAF6 in gliomas [20]. In fact, this miRNA has been reported to be a tumour-suppressor molecule, or conversely, an oncomiR, in many various cancer types [21]. In bladder cancer, it is usually upregulated, and this event is correlated with the inhibitory effect on the invasion of bladder cancer that resulted from the reduction of the matrix metalloproteinase MMP2 expression. It has also been proven that miR-146b knock-down attenuated ETS2 expression in cell lines and in mice, with ETS2 being the significant transcription factor for the expression of MMP2 [21].

Its family ‘cousin’, miR-146a, is known to be involved in bladder cancer relapse, affecting the function of bladder cancer stem cells both directly and indirectly [22]. It was also shown that miR-146a is important for the maintenance of breast cancer stem cells during the epithelial–mesenchymal transition (EMT) by suppressing the expression of the Notch signalling inhibitor NUMB [23]. It has already been proposed that miR-146a-5p levels measured in the urine samples of patients might be used as a prognostic marker for bladder cancer [22,24]. Interestingly, miR-146a—our fourth-best hit—is known to mediate the suppression of inflammatory response in adipocytes [25], which suggests that it might have a similar effect in cases of cancer disease. The expression of the third-best hit, miR-142-3p, was reported to be linked with reduced regulatory T-cell function in granulomatosis [26]. It also suppresses cell proliferation and cell migration in bladder cancer [27]. These and other miRNAs found to be the most correlated with the expression of checkpoint genes are described in Table 1.

Correlation with the expression of several other miRNAs have been noted, but with surprisingly low statistical significance, given the information from the existing papers, for example, high miR-34a expression sensitised MIBC to cisplatin, also inhibiting tumorigenicity and cancer cells proliferation [76]. Connection with epigenetic changes was also observed and reported in the literature: cisplatin-based therapy induces demethylation of miR-34a promoter region, and thus, increases its expression [76]. Despite the significance of miR-34a in bladder cancer and suggested role in checkpoint immune mechanisms, it seems unrelated to the expression of checkpoint genes per se.

### 3.2. The Most Anti-Correlated miRNAs

Our top hit in anticorrelated miRs, mir−200a, is known to often play a role in cancer. However, it is reported to regulate the EMT rather than immune response. It is also predictive for prognosis in colorectal cancer patients. Specifically, in bladder cancer, it was reported to be correlated with early-stage and T1 bladder tumour progression [77] and bladder cancer invasion [78]. Overexpressed miR-200a is known to promote bladder cancer invasion through the direct regulation of the axis Dicer/miR-16/JNK2/MMP-2E [78]. Moreover, miR-200a is very important in ovarian carcinoma: it promotes cell invasion and migration by targeting PTEN. The only reports of mir-200a involvement in the immune response to date come from small airway epithelial cells lung cancer [79].

Although the role of hsa-miR-200b has not been clear in bladder cancer, it seems particularly significant in renal cancer: it is often downregulated and may suppress metastasis by targeting *LAMA4* in renal cell carcinoma [80]. The significantly aberrant expression has also been reported in HER-2 negative breast cancer [81], as well as cardiological pathologies and angiogenesis aberrations [82,83]. In breast cancer, it has also been reported that miR-200b may affect breast cancer cells’ response to tamoxifen, involving MYB [84]. Moreover, it has been suggested as a prognostic marker in clear cell renal carcinoma [85]. Finally, epigenetic silencing of miR-200b was connected with cisplatin resistance in bladder cancer [86]. 

miR-200c seems to be particularly important in breast cancer pathogenesis and response to treatment; for example, several authors indicated its role in cancer cell sensitivity to therapy, including trastuzumab use in HER2 positive breast cancer [87,88]. Interestingly, miR-200c might act protectively against colorectal cancer through the BMI1 gene complex [89]. Furthermore, it suppresses tumour metastasis by inhibiting EMT in oral squamous carcinoma [90]. Although its role in bladder cancer remains unknown, the level of miR-200c in bladder cancer patients’ urine is significantly different from the level in healthy people; thus, it has been suggested as a potential biomarker [32,91].

In our research, miR-200 family seems to be important in the regulation of several checkpoint genes, especially CD48, CD70, CD80, CD86 and PD-L2, as well as transcription factor BACH2, which is known to be involved in transcription regulation of these genes. This finding suggests that it might be a strong candidate for future investigations, especially those aiming at biomarker discovery and new drug targets.

These and other significant miRs revealed in this study are described in Table 2.

### 3.3. Other miRNAs Important in Bladder Cancer

There are several miRNAs described extensively in the bladder cancer literature, but surprisingly, they are not significantly correlated with any of the analysed genes. For example, miR-497 was significantly downregulated in bladder transitional cell carcinoma cells and tissues [8]. This led to the upregulation of the transcription factor E2F3 and suppression of cell proliferation and invasion [8]. On the other hand, the transcription factor E2F has been shown to regulate the expression of miR-15b, a member of the same miR family as miR-497 [137]. Similarly, miR-106a had an inhibitory effect on the proliferation of BC cells through the modulation of MAPK signalling [8].

Several authors have already emphasised the importance of immune cells infiltrations in the tumour as a key component of the patient’s response to PD-1/PD-L1 checkpoint inhibition. Additionally, the presence of specific cells and/or components in the cancer microenvironment can be crucial, for example, in myeloid cells. This phenomenon was observed not only in melanoma or bladder cancer, but also in other not-so-well-investigated cancer types in terms of immunotherapy, such as pancreatic cancer [178]. MicroRNAs have been shown to be important in the patient’s response to therapy. For instance, in chemoresistant ovarian cancer, miR-424 has been proved to regulate the PD-1/PD-L1 and CTLA-4/CD80 pathways [179]. The expression of miR-424 is inversely correlated with the expression of PD-1, PD-L1, CTLA-4 and CD80 genes, and it has been shown that miR-424 inhibited the expression of those genes through direct binding to the 3’UTRs [99]. Moreover, the progression-free survival (PFS) of ovarian cancer patients has been positively correlated with high levels of this miR [179].

### 3.4. Limitations to the Research Presented

As in any research, there are several limitations to our work which need to be mentioned. First, our results are based on the retrospective analysis of a single dataset of 402 cancer samples. The correlations should, therefore, be verified using other data cohorts. Secondly, our analysis is based on the expression data. The RNA-seq data do not measure the final concentration of protein in the cytoplasm and at the cell surface. With a comprehensive proteomics dataset, a more in-depth analysis of actual protein levels and protein–protein interactions at the cancer cell membrane could be performed. This is especially important for the surface proteins of the immunological synapse.

## 4. Materials and Methods

### 4.1. Data Acquisition and Pre-Processing

We acquired miRNA expression, mRNA expression and clinical metadata for cancer cohorts from The Cancer Genome Atlas (TCGA) database using the “TCGAbiolinks” package in R (see Supplementary Materials Section). For correlation analyses, we used the results of “HTSeq - FPKM” workflows for mRNA and read counts for miRNA. We discarded duplicates of the same patient and selected only for experiments where the matching sample was profiled for gene expression and miRNA expression. Of note, healthy tissue samples marked as “controls” were derived from the same patients from which the tumour samples were derived.

### 4.2. Correlation Analyses

Based on the literature, 21 genes potentially expressed at cancer/APC side of the immunological synapse were selected for further analyses. We selected mRNAs of interest and created an n × m numeric matrix of expression measures representing reads per million per kilobase of transcript (RPKM) for RNA-seq, where n is the number of individual samples and m is the number of pre-selected mRNAs. In a similar manner, we created a matrix presenting microRNA expression values—an n × k numeric matrix of expression values, where n is the number of individual samples and k is the number of miRNAs.

We then generated a nonparametric correlation matrix between miRNA and mRNA expression values using Spearman’s rank-order correlation coefficient implemented in R. The statistical significance of each correlation is determined using a correlation test: a t-test is applied to the individual correlations using the following formula: t = r* sqrt(n-2)/sqrt(1-r^2). This method is implemented in the “psych” package. The *p*-values are then corrected to q-values using the false detection rate (FDR) method [179]. The resulting correlations and associated q-values are visualised for further inspection using a custom correlation heatmap drown in “GGplot2” package. Because of the large number of miRNAs, we also provide interactive heatmaps drown using “d3heatmap” package. All interactive and full static heatmaps are available in the Supplementary Materials Section. For figures shown in the manuscript, in the interest of clear visualisation, we have filtered out miRNAs with only non-significant or low correlations to 21 re-selected mRNAs.

### 4.3. Graphical Model Estimation for Correlation Networks

Correlation graphs represent the correlation matrix with nodes that indicate genes of interest and edges that represent correlation values. Green edges indicate positive correlations and red edges negative ones. The width of the edges and their colour saturation corresponds to the absolute value of correlations and scale relative to the strongest weight in the graph. The graphs are organised as “spring” layout, which uses the Fruchterman–Reingold algorithm [11] to obtain a force-directed layout. In this solution, each node (connected and unconnected) repulses the other, while connected nodes attract each other. After a number of iterations (500), a final logout is reached—the distance between the nodes corresponds well to the correlation between the nodes, where correlated nodes are close to each other, while anticorrelated (negative correlation) nodes are moved to distant parts of the graph.

## 5. Conclusions

Our results clearly indicate several miRNAs that might be important in post-transcriptional checkpoint genes regulation on tumour side of the immunological synapse. Moreover, some microRNAs might indirectly participate in transcriptional regulation of checkpoint genes in bladder cancer. Further experimental research is required to confirm and quantify these interactions. MicroRNAs acting as moderators of immune response at the cancer side of the synapse are promising drug candidates and biomarkers. They can be used directly as novel biologics to modulate the synapse, aiming to increase tumour immunogenicity, and thus, as the response to the immune checkpoint inhibitor therapy in bladder cancer. Despite our better understanding of immune response regulation and new checkpoint inhibitor therapeutics being introduced to the market, cisplatin-based chemotherapy remains the major therapeutic agent used to treat bladder cancer worldwide [5]. Only about half of patients respond to this treatment, and eventually, all patients develop a chemotherapy resistance [5,180]. Therefore, we see a crucial need for finding new, highly individualised therapeutic strategies together with molecular biomarker-based companion diagnostics. MicroRNAs play a crucial role in many, if not most, of the processes leading to the development, growth, invasiveness and progression of the tumour. Given their favourable molecular characteristics and ease of detection, they are promising biomarker and therapeutic target candidates. We postulate that further research into the function of microRNA in cancer will yield predictive and prognostic biomarkers to be used in rapid, inexpensive and accurate diagnostics [14].

## Figures and Tables

**Table 1:** The most positively correlated miRNAs and their known functions in human cancers, including bladder cancer, if such a function was reported.

miRNA	Function	References
hsa-miR-21	Impact on survival and prognosis in patients with pancreatic cancer; exosomal miR-21 promotes proliferation, but also invasion and therapy resistance of colon adenocarcinoma cells via its target PDCD4; nothing is known about its role in bladder cancer; a lack of miR-21 expression in tumor-associated macrophages (TAMs) promotes antitumoral immune response.	[28,29,30]
hsa-miR-29a	Downregulated in bladder cancer; shows inhibition of proliferation in bladder cancer cell lines via PI3K-AKT pathway; acts as a tumour suppressor in many cancer types; increased urine levels correlated with shorter event-free survival in most cancer types; high expression of miR-29a was associated with a prolonged disease-free survival.	[31,32,33,34,35]
hsa-miR-100	Suggested role in the invasion and metastasis of hepatocellular carcinoma; involved in the PI3K/AKT and mTOR pathways in renal carcinoma; interestingly, a variant in the miR-100 gene is a protective factor of childhood acute lymphoblastic leukaemia.	[36,37,38]
hsa-miR-125b	Suppresses cell proliferation and metastasis by targeting the *HAX-1* gene in esophageal squamous cell carcinoma; regulates IL-1β-induced inflammatory genes through targeting TRAF6-mediated MAPKs and NF-κB signaling in human osteoarthritic chondrocytes; acts as an oncogene in glioblastoma cells and inhibits cell apoptosis through p53 and p38MAPK-independent pathways; has a role in conferring the metastatic phenotype among pancreatic cancer cells; unknown function in bladder cancer.	[NO_PRINTED_FORM][39,40,41,42]
hsa-miR-142	Linked to the reduced regulatory T-cell function in granulomatosis; suppresses cell proliferation and cell migration in bladder cancer.	[25,26]
hsa-miR-146a	Mediates the suppression of inflammatory response in adipocytes; involved in bladder cancer relapse; important for the maintenance of breast cancer stem cells during EMT; suggested that the urine levels might be possibly used as a prognostic marker for bladder cancer; in bladder cancer, it is usually upregulated, and this event is correlated with the inhibitory effect on the invasion of cancer cells resulting from the reduction of *MMP2* expression.	[20,21,22,23,24,25]
hsa-miR-150	Acts as a tumour promoter: promotes cell proliferation, migration and invasion of cancer cells through targeting PDCD4 (programmed cell death 4 protein); modulates cisplatin chemosensitivity and invasiveness of muscle-invasive bladder cancer cells via targeting PDCD4; is suggested as a urinary biomarker for bladder cancer progression; its agonist promotes fibrosis in cultured kidney cells, while its antagonists decrease pro-inflammatory cytokines and pro-fibrotic proteins and increase anti-fibrotic protein SOCS1.	[43,44,45,46,47]
hsa-miR-155	Tumour-promoting and highly oncogenic microRNA that targets ELK3 transcription factor functioning in the hypoxia response; upregulated in breast cancer and linked to PARP-1 inhibitors response; overexpressed in bladder cancer, promoting tumour growth by repressing *DMTF1.*	[17,18,19]
hsa-miR-199a	Functions as a tumour suppressor in oral squamous cell carcinoma, targeting the IKKβ/NF-κB signalling pathway; inhibits malignant progression of lung cancer through mediating RGS17; serum levels were suggested as a potential diagnostic biomarker for detection of colorectal cancer; recently discovered to inhibit angiogenesis by targeting the VEGF/PI3K/AKT signalling pathway in an in vitro model of diabetic retinopathy; can attenuate aerobic glycolysis and cell proliferation in glioblastoma, but the role in bladder cancer remains unrevealed.	[48,49,50,51,52] [NO_PRINTED_FORM]
hsa-miR-199b	Downregulated in breast cancer patients; is often associated with malignant clinical characteristics; exerts tumour suppressive functions in hepatocellular carcinoma by targeting JAG1 directly; suppression of miR-199b expressions improves apoptosis and reduces the cell viability in cervical cancer.	[53,54,55,56]
hsa-miR-221	High expression is a poor predictor for glioma; affects proliferation and apoptosis of gastric cancer cells (through targeting SOCS3); promotes cisplatin resistance in osteosarcoma cells by targeting PPP2R2A; the function in bladder cancer is unknown.	[57,58,59]
hsa-miR-223	Tumour-suppressive, but also oncogenic miR in various cancers; targets WDR62 directly in bladder cancer—the knockdown of *WDR62* in mice significantly inhibited tumour aggressiveness and induced the apoptosis of bladder cancer cells; it may also inhibit migration and invasion of bladder cancer cells.	[60,61,62,63,64]
hsa-miR-511	circZFR promotes cell proliferation and migration by regulating the miR-511/AKT1 pathway in hepatocellular carcinoma; promotes proliferation of human hepatoma cells; functions as a tumour suppressor and a prognostic marker in colorectal cancer; contributes to intestinal inflammation; significantly altered expression and its target *AKT3* have negative prognostic value in prostate cancer, but its function in bladder cancer remains unknown.	[65,66,67,68,69]
hsa-miR-4772	Significance in bladder cancer and immunological surveillance remains unclear, but a high level in serum exosomes derived from stage II and stage III colon cancer patients was negatively associated with the risk of recurrence and the risk of death.	[70,71]
hsa-miR-5586	Downregulated in pancreatic and bladder cancers; high levels linked to good outcomes in diffuse large B-cell lymphoma (DLBCL); significance in bladder cancer and immunological surveillance remains unknown.	[72,73]
hsa-miR-7702	Potentially important in colorectal cancer (CRC) progression, but significance in bladder cancer remains unclear.	[74,75]

**Table 2:** The most negatively correlated miRNAs and their known functions in human cancers, including bladder cancer, if such a function is reported.

miRNA	Function	References
hsa-miR-30d	Involved in suppressing endoplasmic reticulum and chaperone and signalling regulators in several human cancers; suggested as a tumour supressor in lung cancer initiation and progression.	[92,93,94,95,96]
hsa-miR-93	Probably associated with the prognosis of bladder cancer; known to promote bladder cancer cells proliferation and invasion via targeting *PEDF* gene; involved in sensitivity of bladder cancer to chemotherapy; promotes hepatocellular carcinoma progression, possibly via miRNA-93-5p/MAPK/c-Jun positive feddback loop.	[97,98,99,100,101,102]
hsa-miR-96	Oncogenic miR; potential urinary biomarker in bladder cancer; involved in EMT and migration and invasion of bladder cancer cells via targeting CDKN1A; impacts the response to chemotherapy.	[103,104,105,106,107,108]
hsa-miR-98	Important in the development and progression of bladder cancer due to its involvement in the WNT/β-catenin pathway; promotes drug resistance via targeting the *LASS2* gene; axis miR-98/IGF1 contributes to breast cancer progression.	[109,110,111,112,113]
hsa-miR-141	Might be important in the wound-healing process; promotes bladder cancer progression, and thus, has prognostic value; in oesophageal cancer, promotes cell proliferation, migration and invasion; moreover, in ameloblastoma, it has been shown to supress cell migration via upregulation of the NCAM1 molecule.	[114,115,116,117]
hsa-miR-151a	Studied in atopic dermatitis, pain transmission and NOTCH2 signalling pathway; targeting CHL1 inhibits proliferation and invasion of colon cancer cells; in nasopharyngeal carcinoma, the inhibition of p53 by miR-151a induced cell proliferation, migration and possibly also invasion.	[118,119,120,121]
hsa-miR-182	Potentially important in the bladder cancer development, proliferation and migration; inhibits inflammation, proliferation and migration of endometrial stromal cells through NFkB pathway deactivation.	[122,123,124,125]
hsa-miR-183	Involved in the cell adhesion modulation and progression of laryngeal cancer; reports in bladder cancer indicated its crucial role in maintaining the canonical WNT signalling pathway, regulating growth and apoptosis; it has also been reported that cisplatin and paclitaxel significantly alter the expression of miR-183; potentially involved in hepatocellular carcinoma cells proliferation by LNC-HC gene inhibition.	[108,126,127,128,129]
hsa-miR-187	Oncogene miR known to be involved in proliferation, migration, invasion and recurrence bladder cancer; regulates the WNT/β-catenin pathway.	[130,131]
hsa-miR-191	Linked to the antiviral response and intracellular mechanisms of viral replication, e.g., may inhibit the replication of human immunodeficiency virus in human cells; circulating miR-191 has been proposed as a biomarker of breast cancer, as well as squamous cell carcinoma; it has been suggested to regulate endometrial cancer cell growth via TET1-mediated epigenetic modulation of APC gene.	[132,133,134,135]
hsa-miR-200a	Regulation of EMT; probably predictive for a patient’s prognosis in colorectal cancer; reported to be correlated with early-stage and T1 bladder tumour progression and bladder cancer invasion; when overexpressed, it promotes bladder cancer invasion.	[77,78]
hsa-miR-200b	Significant in renal cancer: it is often downregulated and may suppress metastasis by targeting *LAMA4* in renal cell carcinoma; aberrant expression has been reported in HER-2 negative breast cancer as well as cardiological pathologies and angiogenesis aberrations; may affect breast cancer cells’ response to tamoxifen, involving MYB; it has been suggested as a prognostic marker in clear cell renal carcinoma; epigenetic silencing of miR-200b is connected with cisplatin resistance in bladder cancer.	[83,84,85,136,137,138]
hsa-miR-200c	Important in breast cancer pathogenesis and response to treatment, including trastuzumab use in HER2 positive breast cancer; might act protectively against colorectal cancer through BMI1 gene complex; it suppresses tumour metastasis by inhibiting EMT in oral squamous carcinoma; the role in bladder cancer remains unknown—the level of miR-200c in bladder cancer patients’ urine is significantly different from the level in healthy people, and thus, it has been suggested as a potential biomarker.	[32,87,88,89,90,91]
hsa-miR-219a	Inhibits colon cancer progression; enhances the radiosensitivity of lung cancer cells.	[139,140]
hsa-miR-301b	Accelerates the growth of gastric cancer; promotes the mobility, proliferation and EMT in bladder cancer by targeting EGR1; plasma levels of miR-301b might be a potential biomarker of early stage non-small-cell lung cancer.	[141,142,143,144]
hsa-miR-423	It has been proposed as a biomarker, indicating early stages of bladder cancer, especially from blood serum and urine; suppression of miR-423 in breast cancer cells inhibited cell proliferation and invasion.	[46,145,146,147]
hsa-miR-429	Known to be involved in the pathogenesis of many cancer types, including bladder; significant role in EMT in bladder cancer; expression levels have been correlated with patient outcomes; it seems to promote proliferation of bladder cancer cells via the inhibition of CDKN2B; elevated miR-429 supresses the progression of hyphopharyngeal squamous cells carcinoma by reducing ZEB1 expression.	[148,149,150,151,152]
hsa-miR-744	Although its function in bladder cancer has to be revealed, it seems important in colorectal cancer, ovarian cancer and heart diseases, targeting the ARF1 gene; interestingly, it has been reported to be involved in regulation of the MHC class I gene expression; contributes to inflammation among patients with Sjorgen Syndrome; promotes proliferation of osteosarcoma cells by targeting PTEN.	[153,154,155,156,157]
hsa-miR-940	Known to impact the aggressiveness of bladder cancer cells via activating the WNT/β-catenin pathway; suggested as a biomarker of gastric cancer; involved in the hepatocellular cancer and prostate cancer development.	[158,159,160,161]
hsa-miR-1287	Probably regulates the MEK/ERK pathway; the role in immune surveillance remains unknown.	[162,163]
hsa-miR-1306	Connected with the SIRT gene family expression; important in gastric cancer; its role in immune surveillance is to be revealed.	[164,165,166]
hsa-miR-1307	Upregulation contributes to the progression of gastric cancer; indicates the metastatic potential of hepatocellular carcinoma; by targeting TRAF3 gene, it also regulates the MAPK/NFkB pathway in lung adenocarcinoma, promoting cancer cells’ proliferation.	[167,168,169]
hsa-miR-3193	Very little is known about this miR; however, its role has been reported in melanoma pathogenesis.	[170]
hsa-miR-3200	Acting in gastric cancer tumorigenesis and progression.	[171,172,173]
hsa-miR-4756	Seems to be particularly important in triple-negative breast cancer pathogenesis; also reported in the EMT process.	[174,175]
hsa-miR-4778	Might be involved in radioresistance development in cervical cancer.	[176]
hsa-miR-7706	Little is known about this miR and its function in cancer pathogenesis; in hepatocellular carcinoma, it seems to have a prognostic value.	[177]

## Data Availability

Not applicable.

## Supplementary Materials

Figure S1: Heatmap showing all corelations between selected gens and miRNAs as PDF; Figure S2: Interactive heatmap showing all corelations between selected gens and miRNAs—online resource at https://przemol.github.io/blca/ [181,182].
